# ADHD traits and financial decision making in stock trading

**DOI:** 10.1038/s41598-025-17467-3

**Published:** 2025-08-31

**Authors:** Max Witry, Marcel Schulze, Niclas Braun, Henrik Rohner, Johannes Weller, Philipp Müller, Alexandra Philipsen, Markus Kölle

**Affiliations:** 1 Department of Psychiatry, University Hospital Bonn, Venusberg Campus 1, 53127 Bonn, Germany; 2 Department of Vascular Neurology, University Hospital Bonn, Venusberg Campus 1, 53127 Bonn, Germany; 3 Department of Neuro-Oncology, University Hospital Bonn, Venusberg Campus 1, 53127 Bonn, Germany

**Keywords:** Diseases, Medical research, Psychology, Psychology, Risk factors

## Abstract

Attention-Deficit/Hyperactivity Disorder (ADHD) is a common neurodevelopmental condition characterised by inattention and impulsivity. While its effects on academic and occupational functioning are well established, less is known about how ADHD traits relate to financial behaviours, particularly in high-risk contexts such as online trading. This cross-sectional study examined associations between ADHD traits and financial decision-making in a sample of 945 active online traders. Participants were assessed using a validated self-report scale for ADHD traits and standardised measures of financial risk tolerance (FRT), trading activity, and portfolio performance. The median participant age was 34 years, with 74.5% identifying as male. Based on self-report, 5.7% of participants met DSM-5 symptom criteria suggestive of ADHD, though no clinical diagnosis was made. Participants with ADHD traits demonstrated significantly higher FRT and greater speculative risk-taking. ADHD traits correlated positively with FRT and negatively with portfolio returns. Regression analyses revealed that increased trading frequency, speculative risk-taking, and higher return expectations predicted greater ADHD trait expression, while higher actual portfolio returns and higher investment risk scores were associated with lower ADHD traits. Though causality cannot be inferred, traits like inattention and impulsivity might contribute to suboptimal decision-making in volatile financial settings. Clinicians should consider this in their assessments, promote awareness and support prevention of poor investment decisions. Further longitudinal studies are needed, alongside targeted financial guidance for individuals with elevated ADHD traits.

**Supplementary Information:**

The online version contains supplementary material available at 10.1038/s41598-025-17467-3.

## Introduction

Attention-deficit/hyperactivity disorder (ADHD) is a neurodevelopmental disorder characterised by persistent inattention, hyperactivity and impulsivity, which affects both children and adults^1^. ADHD presents in predominantly inattentive, predominantly hyperactive-impulsive, and combined presentations, with symptoms existing on a spectrum where subclinical traits can cause significant impairment despite not meeting full diagnostic criteria^2^. The global prevalence of adult ADHD is estimated at 2.6%^3^, with significant implications for academic, occupational and social functioning^4^. Adults with ADHD are at increased risk for financial difficulties, such as impulsive spending, accumulating debt and challenges with saving^5^. Compared to individuals without ADHD, they often have lower lifetime earnings and greater financial dependence on family and public welfare^6^. Research by Beauchaine et al.^7^ further highlights the extent of these difficulties, demonstrating that ADHD symptoms are associated with delay discounting and a range of adverse financial behaviours, including late credit card payments, reliance on high-interest borrowing and pawn services, personal debt, and unstable employment histories. Additionally, financial decision-making in individuals with ADHD is often marked by maladaptive behaviours, including impulsive purchases and decision avoidance^8^. Moreover, research suggests that ADHD is associated with heightened temporal discounting^9^favouring immediate rewards over delayed benefits, which may contribute to increased financial risk tolerance and more speculative investment behaviour^10^. Consequently, impulsivity and overconfidence can further exacerbate decision-making challenges, leading to suboptimal financial outcomes^11^. A study has shown that adults with ADHD exhibit impairments in decision-making tasks that require substantial cognitive control, where both deliberative and affective systems are involved^12^. These impairments tend to be most pronounced in individuals with ADHD-Combined presentation^13^.

Financial risk tolerance (FRT), influenced by factors such as sex, income and wealth^14,15^is a key determinant of investment behaviour, with higher degrees indicating greater risk taking. Men, higher-income earners and wealthier individuals generally exhibit higher FRT, whereas life events like marriage and parenthood typically reduce risk-taking behaviour^16^. The Grable and Lytton questionnaire consists of subscales assessing different aspects of FRT, such as the speculative, gambling and investment risk scales^15^. The speculative risk scale reflects short-term, high-variance financial behaviour, which is often associated with traits like thrill-seeking and delay aversion^15^. The gambling risk scale assesses how well individuals evaluate trade-offs between certain returns and uncertain, probabilistic gains; whereas the investment risk scale integrates risk tolerance by combining financial knowledge and emotional resilience^15^.

Individuals with ADHD often demonstrate more impulsive and spontaneous financial behaviours, associated with poorer financial outcomes and potentially greater financial risk-taking^17^. Recent research found that adults diagnosed with ADHD engage in significantly higher levels of financial risk-taking in areas such as investment and gambling compared to healthy controls^18^. This underscores the critical need for targeted interventions to improve financial decision-making in individuals with ADHD.

While most research has focused on individuals with clinically diagnosed ADHD, this study extends existing work by examining ADHD traits within a sample of active traders. We hypothesise that increased FRT, indicating riskier financial decisions, will be associated with higher ADHD traits (i.e. inattention as well as hyperactivity/impulsivity). An online survey was conducted to assess ADHD traits and FRT through validated self-report instruments, alongside data collection on participants’ financial behaviours. This study offers new insights into the financial behaviours of individuals with ADHD traits, distinguishing it from previous research on clinically diagnosed individuals.

## Methods

### Study design and participants

This cross-sectional study was conducted via an online survey from September 5 to November 7, 2024, targeting German-speaking active traders. Participants were primarily recruited through *Getquin*, an investment app, and financial forums from *finanzen.net* and *Akademischer Börsenkreis Universität Halle e.V.*. Additional recruitment methods included ADHD-related platforms (*ADHS Deutschland e.V.*, www.ADXS.org*)* and local German journals (*General Anzeiger Bonn*, *Rheinisches Ärzteblatt*).

To encourage participation, 10 premium Getquin accounts were offered as rewards to participants that had been recruited via this platform. The premium accounts were distributed in a lottery-based manner. Prior to data collection, participants were provided with an overview of the study’s purpose, procedures, and data privacy policies and informed consent was obtained.

### Demographic and trading characteristics

Collected demographic data included age, marital status, education level, income and assets. These variables were assessed using questionnaires with predefined response options. Trading behaviours were assessed based on time spent trading, portfolio tracking, transaction frequency and portfolio performance as well as portfolio expectation. These items were developed specifically for this study to capture core aspects of individual trading activity and align with measures used in recent literature^19^. Active trader status was determined based on participants’ responses to key questionnaire items regarding trading frequency, portfolio performance, and portfolio monitoring.

### Assessment of ADHD traits

ADHD traits were evaluated using the 22-item ADHS-Selbstbeurteilungsskala (ADHS-SB)^20^a validated German self-report questionnaire aligned with DSM-5 criteria^1^. This scale assesses inattention, hyperactivity and impulsivity, with responses rated on a 4-point scale (0 = “absent” to 3 = “severe”), resulting in a total score range of 0–54. Participants were classified as “ADHD-positive” if they endorsed ≥ 5 symptoms of inattention and/or hyperactivity-impulsivity ranked as “severe”, with symptom onset before age 12 and significant impairment in daily life. Since formal clinical diagnostic procedure was not conducted, we refer to these scores as ADHD traits rather than symptoms.

### FRT assessment

FRT was measured using the Grable Risk Tolerance Scale^15^with higher scores indicating greater willingness to take financial risks. To allow for a more detailed analysis, all 20 items (see supplementary material) were recorded, and three subscales were calculated:

1. Gambling risk score – Evaluates the ability to assess trade-offs between guaranteed returns and probabilistic gains. Items present scenarios requiring decisions between a certain, lower payout and a higher, uncertain outcome.
2. Speculative risk score – Measures willingness to engage in speculative financial activities, such as taking financial risks with uncertain but potentially high returns.
3. Investment risk score – Assesses risk tolerance in investment decisions, integrating financial knowledge and emotional resilience. Items reflect willingness to invest in higher-risk assets (e.g., equities, real estate) versus safer alternatives.

### Statistical analysis

Data were analysed using SPSS Version 30. Normality of distributions was assessed with the Shapiro-Wilk test. For correlations analysis we used Pearson’s correlation for continuous and normally distributed variables and Spearman’s rank correlation for non-normally distributed or ordinal variables. Independent t-tests compared ADHD-positive and ADHD-negative participants and assessed sex differences. Multiple linear regression was used to identify predictors of ADHD trait scores, with FRT subscores, portfolio returns and expectations, portfolio tracking, and trading frequency entered as independent variables. Assumptions of multicollinearity and residual independence were checked using variance inflation factors (VIF) and the Durbin-Watson statistic. Statistical significance was set at *p* < 0.05.

## Results

### Recruitment and participant flow

**Fig. 1:**
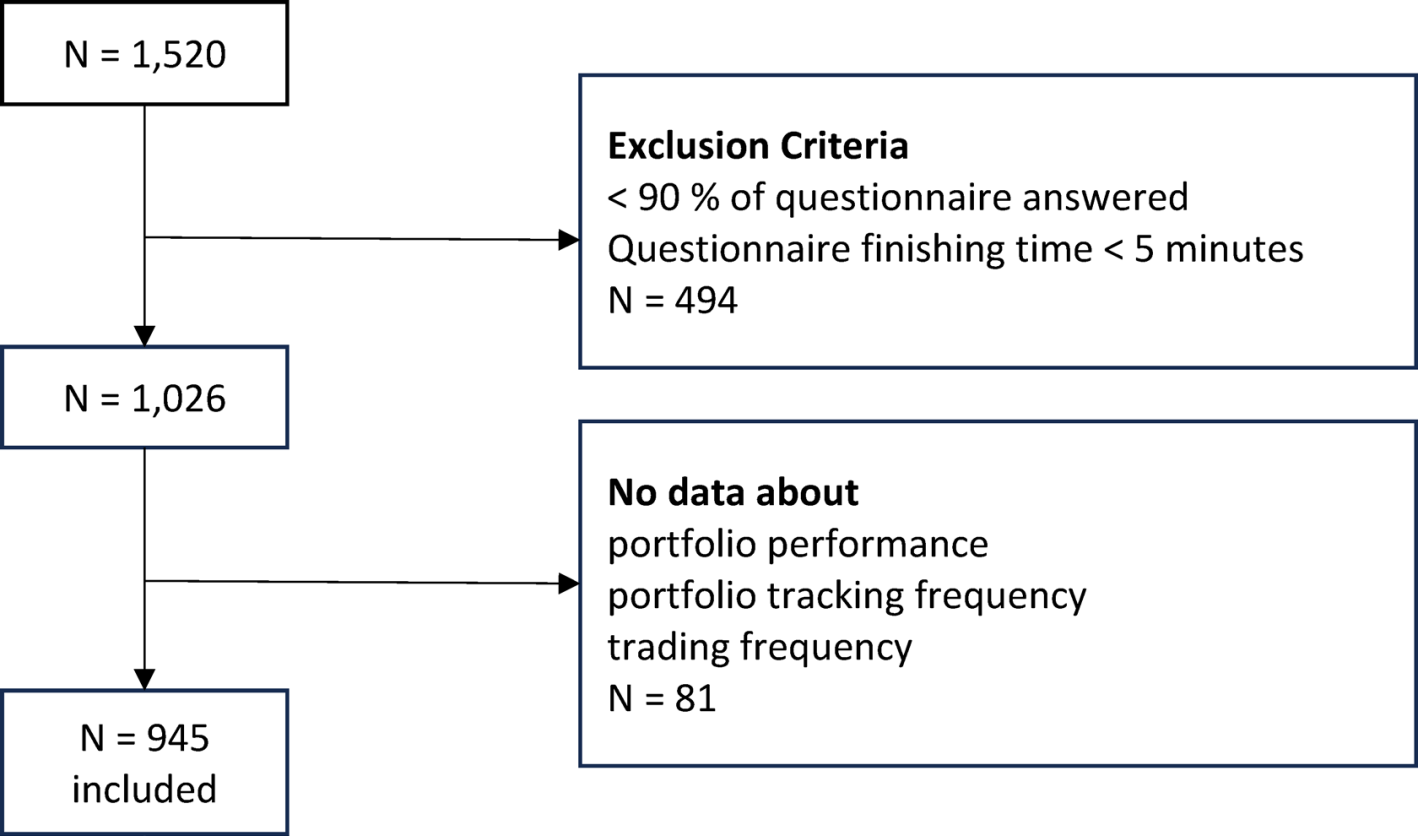
Flowchart of the sample acquisition. Participants of the online survey were recruited via various platforms. Initially, 1,520 people participated. Out of these, 494 were excluded since they answered less than 90% of the questionnaire or finished the survey in less than 5 min. Further 81 participants were excluded from analysis as they did not give key information on their portfolio performance, portfolio tracking and/or trading frequency.

A total of 1,520 participants were recruited, with 945 (62.2%) completing the survey with valid data. The remaining 575 participants (37.8%) were excluded due to incomplete responses, defined as providing less than 90% of the survey, finishing in under five minutes (average completion time was 12 min), or missing key data relevant for characterising trading behaviour, such as trading frequency, portfolio performance and portfolio tracking (see flow chart in Fig. 1).

### Participant characteristics

The mean age of participants was 35.5 years (SD ± 11.95). The sample was predominantly male (74.5%), with 24.9% female and 0.6% identifying as diverse. Marital status was as follows: 37.3% married, 34.8% single, and 62.6% had no children. Educational attainment was high: 66.1% held a high school diploma, 48.0% a university degree, 12.4% a doctorate and 1.7% a postdoctoral qualification. Nearly 60% reported net earnings exceeding €3,000 per month, which is higher than the average income in Germany (*Statistisches Bundesamt*, 2025). Additionally a notably high proportion reported assets over €500,000 (20.3%, see Table 1).

### Financial behaviours

**Fig. 2:**
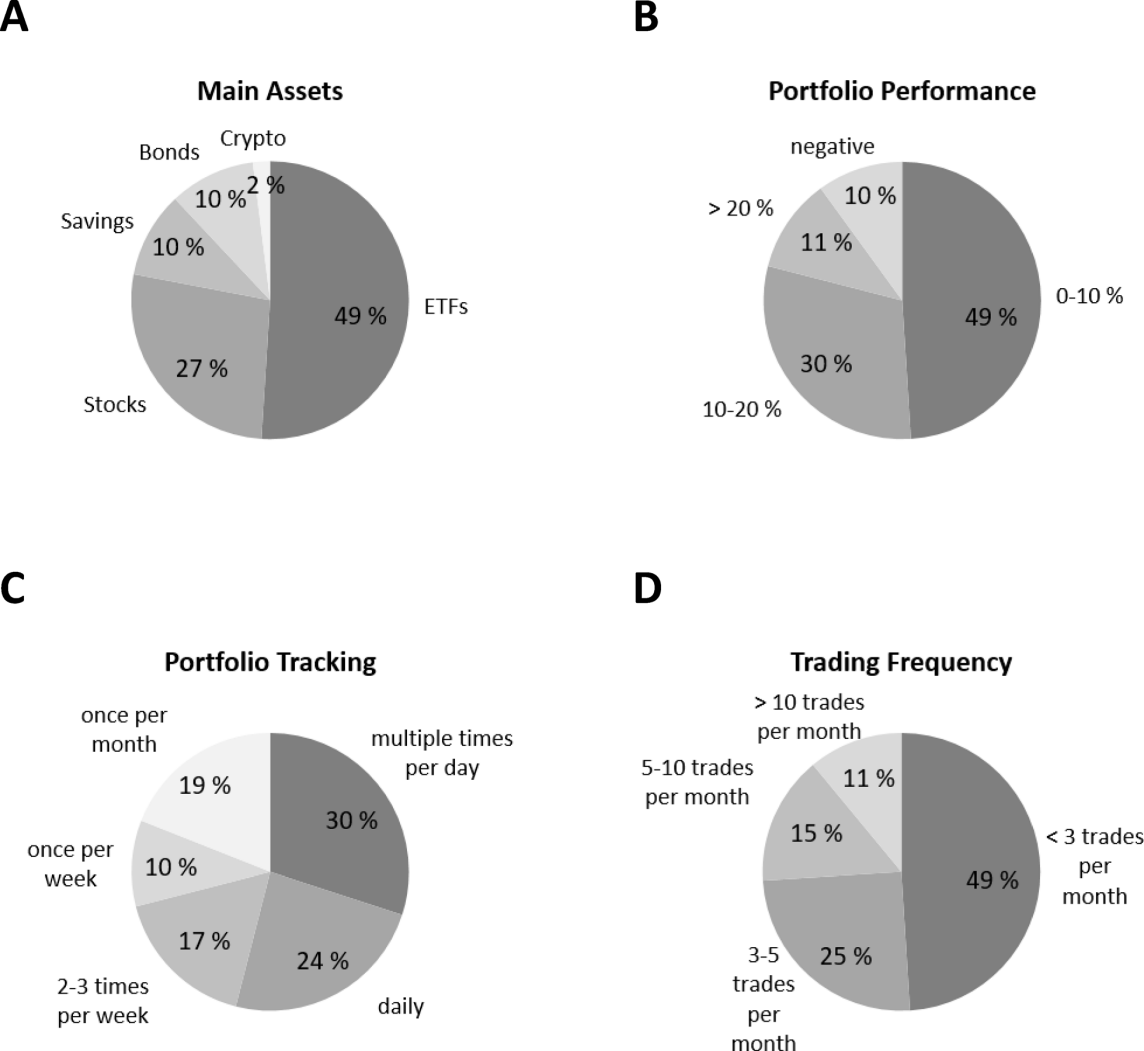
Financial Behaviour of the investigated sample. (**A**) Main assets in investment, (**B**) portfolio performance reported over the past 12 months, (**C**) portfolio tracking and (**D**) trading frequency.

Participants were highly engaged in financial management, with 30.1% checking their portfolios multiple times daily. Preferred investments included exchange-traded funds (ETFs, 50.6%), individual stocks (26.5%) and real estate (9.9%). Most participants (90%) reported positive portfolio returns in the past year: 50.4% earned 0–10%, 30.0% earned 10–20% and 10.9% earned > 20%. Regarding trading frequency, 75% of participants traded fewer than five times per month, while 5% traded over 10 times and another 5% traded more than 20 times monthly (see Fig. 2).

### Prevalence of ADHD traits

The median ADHD symptom score in the ADHS-SB was 17.53 (SD ± 13.9, range: 0–54). While a total score of ≥ 18 is considered a suggestive threshold^20^classification in this study was based on DSM-5 criteria mirrored through ADHS-SB subscale responses. According to DSM-5 criteria^1^54 participants (5.7%) were classified as ADHD-positive. Of these, 30 (56%) exhibited primarily inattention symptoms, 17 (31%) exhibited hyperactivity/impulsivity and 7 (13%) showed a combined presentation. Notably, 17 of the 54 ADHD-positive participants (31%) were female, a slightly higher proportion than in the overall sample (24.9%). Additionally, females reported significantly higher ADHD symptom scores than males (19.1 vs. 15.9; *p* < 0.01). Finally, ADHD traits showed small but significant negative correlations with age, education, income, and assets (see supplementary Table 1).

### FRT in ADHD

**Fig. 3:**
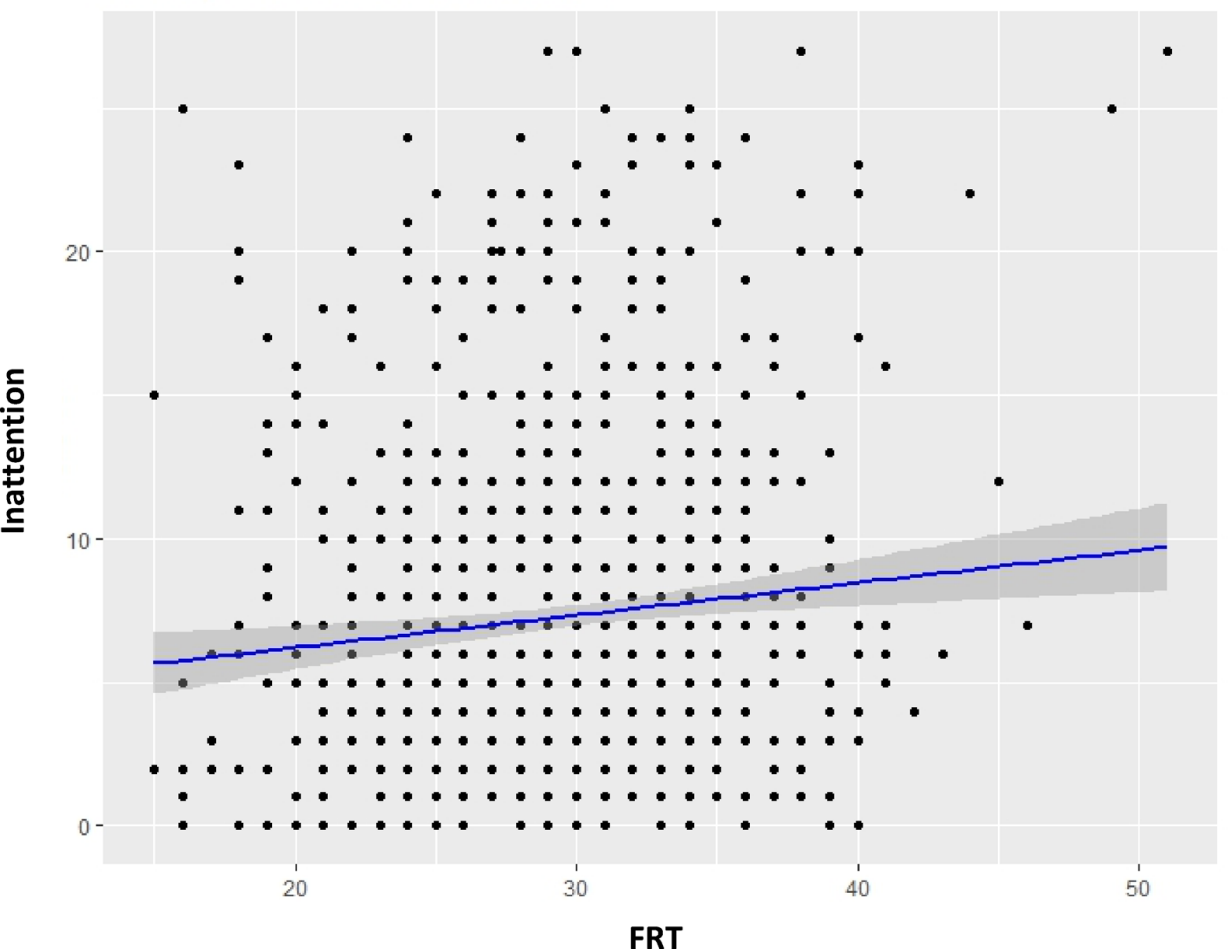
Correlation between Inattention score and Financial Risk Tolerance (FRT). Each point represents an individual participant’s scores. *r* = 0.103, *p* = 0.01.

FRT scores averaged 29.5 (SD ± 5.9, range: 0–47) and were normally distributed, with 22% of participants scoring ≥ 33, classified as highly risk-tolerant. Those in the ADHD-positive group had significantly higher mean FRT scores compared to the ADHD-negative group (30.9 vs. 29.4; *p* = 0.025), driven mainly by greater speculative risk-taking (11.6 vs. 10.9; *p* = 0.015), while no significant differences emerged for gambling or investment risk scores. ADHD traits were positively correlated with overall FRT (*r* = 0.079, *p* = 0.015), particularly with inattention symptoms (*r* = 0.103, *p* = 0.01, see Fig. 3) but not with hyperactivity/impulsivity. Additionally, ADHD traits were negatively correlated with portfolio returns over the past 12 months (*r* = -0.075, *p* = 0.02). Significant sex differences were also observed: males demonstrated higher mean FRT scores (30.4 vs. 26.8; *p* < 0.01) and achieved better portfolio returns (4.5% vs. 4.1%; *p* < 0.01). Age, education and income each showed small but significant positive correlations with FRT, whereas assets were not significantly related to FRT. A full matrix of first-order correlations between the study variables is provided in the supplementary material (see supplementary figure and Table 1).

### Regression analysis

Multiple linear regression analysis identified predictors of ADHD symptom scores. Higher trading frequency (B = 0.769, *p* = 0.046), increased speculative risk-taking (B = 0.675, *p* = 0.034), and optimistic portfolio return expectations (B = 1.237, *p* = 0.041) were significant positive predictors. Conversely, higher investment risk scores (B = -0.590, *p* = 0.034) and greater portfolio returns over the past 12 months (B = -1.067, *p* = 0.019) were significantly associated with lower ADHD symptom scores (see Table 2).

## Discussion

In this study we aimed to assess a potential influence of ADHD traits on key financial behaviours and outcomes in a community of active online traders. The cohort, predominantly male (74.5%) with a median age of 34 years, showed above-average levels of education, income, and financial engagement compared to the general population^14,15^. The prevalence of participants screening positive for ADHD (5.7%) was slightly higher than population estimates (2.2–2.8%)^21^, though many individuals exhibited subclinical ADHD traits. In contrast to clinical populations^21^our sample showed a higher proportion of participants with a predominantly hyperactive/impulsive presentation. This unexpectedly high rate may reflect sample-specific bias and is likely influenced by the relatively small number of ADHD-positive individuals.

Our findings in the correlation analysis suggest that ADHD traits go along with higher FRT. Notably, considering the ADHD traits, particularly inattention and not hyperactivity/impulsivity were linked to FRT. At first sight, this may seem counterintuitive, as impulsivity is typically seen as the trait most closely aligned with risk-taking^13^. However, inattention in this context may reflect an inconsistency between initial planning to final execution, as well as difficulties in maintaining strategic focus and resisting distractions. Therefore, inattention may amplify behavioural biases including loss aversion, overconfidence, and recency bias, potentially impacting decision-making in volatile markets^22–24^. These effects may be further shaped by individual circumstances such as having children, outstanding debts, or a mortgage, which can influence both risk perception and the psychological pressure behind financial decisions^25,26^.

Contrary to expectations, no significant correlation was found between hyperactivity/impulsivity and FRT. Seen together, these findings may indicate that impulsive tendencies alone do not drive risky financial decision-making. Instead, impulsive decision-making appears to be driven more by other factors like personality traits, risk perception and expected benefits^27^. It is also possible that the measure used, which included both hyperactivity and impulsivity items, did not isolate impulsivity sufficiently, potentially obscuring relevant associations^28^. This highlights the need for future studies to incorporate more targeted tools, such as the Barratt Impulsiveness Scale^29^to better capture the facets of impulsivity relevant to financial risk-taking.

Further regression analysis identified both positive and negative predictors of ADHD traits in financial behaviour, revealing a pattern consistent with prior psychological and behavioural research^6^.

One of the positive predictors was speculative risk-taking, aligning with existing literature linking ADHD to an aversion against delay and preference for immediate rewards^30^. Surprisingly, the gambling risk score was not a significant predictor, which was unexpected given its conceptual similarity to speculative risk-taking^31^. In our study speculative risk tolerance, linked to delay aversion^9^was positively associated with ADHD traits, whereas investment risk, reflecting long-term decision-making, was negatively related. This contrasts with findings by Hamurcu et al.^18^who reported such associations in individuals with ADHD.

A further positive predictor of ADHD traits was the trading frequency. This supports previous studies suggesting that frequent trading may reflect maladaptive behavioural tendencies, potentially indicative of addictive patterns^32^. Furthermore, it is well known that frequent trading goes with poorer portfolio returns resulting in poorer long-term investment outcomes^33^.

In addition, higher portfolio expectations were positively associated with ADHD traits. These traits may foster short-term, reactionary trading behaviours that prioritise immediate outcomes over long-term financial planning^34^.

On the other hand, negative predictors were the actual portfolio performance and the investment risk score. The latter, which is linked to financial literacy^14^suggests that better-informed individuals make more calculated and consistent investment decisions^35^. In sensation seeking individuals, the role of emotional regulation should also be considered^36^. Emotional dysregulation, common in ADHD, may intensify tendencies toward impulsive, short-term financial decisions. This interpretation is further supported by a preference for immediate rewards, which is central to speculative risk-taking^37^. By assigning ADHD traits as dependent outcome variables in our regression analysis, we provide key parameters that may help to identify elevated ADHD tendencies in non-diagnosed individuals, particularly in non-clinical contexts.

Furthermore, sex differences were observed. Notably, 31% of the ADHD-positive participants were female, a slightly higher proportion than the overall female representation in the sample (24.9%). Given that females are generally less represented in the trading world^38^this imbalace in our sample is not unexpected. Possibly, the higher ADHD rate among female participants reflects a specific selection effect: women who engage in active trading may be more likely to exhibit ADHD traits, such as impulsivity or sensation seeking. Contrasting with trends in the general population, where women typically adopt more cautious financial strategies and often outperform men^39,40^in our data males showed higher FRT and better portfolio returns. However, ADHD traits in females were associated with lower FRT and poorer portfolio outcomes, highlighting the impact of sex differences in ADHD traits regarding financial decision making.

Of note, our study has several limitations. First, its cross-sectional design precludes causal conclusions. Therefore, longitudinal studies will be necessary to further investigate the influence of ADHD traits on financial behaviours over time and vice versa. Moreover, we here only assessed ADHD traits by self-reporting tools. Even though the applied ADHS-SB questionnaire is a validated German screening tool reflecting the DSM-5 criteria of ADHD, a clinical assessment would provide a higher diagnostic precision. Additionally, the effect sizes observed in this study were relatively small, which limits the strength of the conclusions and underscores the need for replication in more diverse samples. Nevertheless, we view this study as an important initial observation that suggests a potential link between ADHD traits and financial behaviour. Moreover, our sample consisted largely of young, educated, and financially engaged individuals, which may limit the generalisability of the findings. Given that higher ADHD symptoms are often associated with lower income and education in the general population^41^the high socioeconomic status of our sample may have influenced the observed associations. Future investigations are necessary to better understand the broader implications of ADHD traits on financial behaviour.

## Conclusions

This study reveal significant associations between ADHD traits, particularly inattention, and financial behaviours such as increased trading frequency, greater risk tolerance, and more speculative decision-making. While ADHD traits turn out to be linked to a preference for immediate rewards, they also correlate with poorer portfolio returns, suggesting that impaired decision-making - rather than a simple preference for risk - drives suboptimal financial outcomes. Physicians and psychotherapists should address this in their clinical conversations and promote preventive strategies when appropriate. Increasing patient awareness may enhance self-regulation of investment decisions. Therefore, systematic research is necessary to evaluate these effects more rigorously.

**Table 1:** Demographic features of study participants.

Category	Variable	*N*	%
Age (years)	17–25	170	18.0
	26–35	341	36.1
	36–45	237	25.1
	46–55	121	12.8
	56–65	64	6.8
	> 65	20	2.2
Gender	Male	704	74.5
	Female	235	24.9
	Diverse	6	0.6
Marital status	Single	329	34.8
	Partnered	237	25.1
	Married	353	37.3
	Divorced	26	2.8
Children	No children	592	62.6
	1 child	135	14.3
	2 children	155	16.4
	≥ 3 children	63	7.3
Education	No school degree	5	0.5
	Secondary school	34	3.6
	High school diploma	135	14.3
	Technical diploma	146	15.5
	University entrance	625	66.1
Highest degree	No degree	96	10.2
	Vocational degree	262	27.7
	University degree	454	48.0
	Doctorate	117	12.4
	Post-doctorate degree	16	1.7
Income (€, net, per month)	< 1,500	126	13.3
	1,500–3,000	273	28.9
	3,000–4,000	196	20.7
	4,000–6,000	202	21.4
	6,000–8,000	78	8.3
	> 8,000	68	7.2
Assets (€)	< 20,000	116	12.3
	20,000–50.000	173	18.3
	50,000–100,000	151	16.0
	100,000–200,000	140	14.8
	200,000–500,000	171	18.1
	> 500,000	192	20.3

**Table 2:** Linear regression model.

	B	CI	β	t	*p*
Investment risk score	-0.59	-1.4, -0.05	-0.11	-2.12	0.034
Speculative risk score	0.68	0.05, 1.13	0.13	2.13	0.034
Gamble risk score	0.04	-0.44, 0.52	0.01	0.17	0.868
Portfolio expectation	1.24	0.05, 2.42	0.07	2.05	0.041
Portfolio performance	-1.07	-1.96, -0.02	-0.08	-2.35	0.019
Portfolio tracking	-0.43	-1.08, 0.21	-0.05	-1.31	0.190
Trading frequency	0.77	0.02, 1.52	0.07	2.00	0.046

B = unstandardised regression coefficient; CI = confidence interval; β = standardised regression coefficient; t = t-test statistic; p = probability value. The model had an overall fit of *R* = 0.161, R² = 0.026.

## Supplementary Information

Below is the link to the electronic supplementary material.

Supplementary Material 1

## Data Availability

Individual participant-level data is not available due to confidentiality concerns and data-sharing agreements in place. However, the study protocol and statistical analysis plan are available upon request from the corresponding author.

## Abbreviations

ADHD: Attention deficit/hyperactivity disorder
ADHS-SB: ADHS-Selbstbeurteilungsskala‘ (ADHD self-assessment scale)
FRT: Financial risk tolerance
