# Supplementary material for: ADHD traits and financial decision making in stock trading
Source: Sci Rep. 2025 Aug 31;15:32011. doi: 10.1038/s41598-025-17467-3 (PMC12399749; doi:10.1038/s41598-025-17467-3)
Supplement: Supplementary file 1 — Supplementary Material 1 [file 41598_2025_17467_MOESM1_ESM.docx]

**ADHD Traits and Financial Decision Making in Stock Trading**

**Max Witry^1*^, Marcel Schulze^1^, Niclas Braun^1^, Henrik Rohner^1^, Johannes Weller^2,3^**, **Philipp Müller^1^, Alexandra Philipsen^1^, Markus Kölle^1^**^1^ Department of Psychiatry, University Hospital Bonn, Venusberg Campus 1, 53127 Bonn, Germany

^2^ Department of Vascular Neurology, University Hospital Bonn, Venusberg Campus 1, 53127 Bonn, Germany

^3^ Department of Neuro-Oncology, University Hospital Bonn, Venusberg Campus 1, 53127 Bonn, Germany

***Corresponding author:**

Dr. med. Max Witry

Venusberg Campus 1

53127 Bonn
[max.witry@ukbonn.de](mailto:max.witry@ukbonn.de)

**Supplementary Material**

**Financial Risk Tolerance Assessment Items**

In general, how would your best friend describe you as a risk taker?

a. A real gambler

b. Willing to take risks after completing adequate research

c. Cautious

d. A real risk avoider

You are on a TV game show and can choose one of the following. Which would you take?

a. €1,000 in cash

b. A 50% chance at winning €5,000

c. A 25% chance at winning €10,000

d. A 5% chance at winning €100,000

You have just finished saving for a "once-in-a-lifetime" vacation. Three weeks before you plan to leave, you lose your job. You would:

a. Cancel the vacation

b. Take a much more modest vacation

c. Go as scheduled, reasoning that you need the time to prepare for a job search

d. Extend your vacation, because this might be your last chance to go first-class

How would you respond to the following statement? "It’s hard for me to pass up a bargain."

a. Very true

b. Sometimes true

c. Not at all true

If you unexpectedly received €20,000 to invest, what would you do?

a. Deposit it in a bank account, money market account, or an insured CD

b. Invest it in safe high-quality bonds or bond mutual funds

c. Invest it in stocks or stock mutual funds

In terms of experience, how comfortable are you investing in stocks or stock mutual funds?

a. Not at all comfortable

b. Somewhat comfortable

c. Very comfortable

Which situation would make you the happiest?

a. You win €50,000 in a publisher’s contest

b. You inherit €50,000 from a rich relative

c. You earn €50,000 by risking €1,000 in the options market

d. Any of the above—after all, you’re happy with the €50,000

When you think of the word "risk," which of the following words comes to mind first?

a. Loss

b. Uncertainty

c. Opportunity

d. Thrill

You inherit a mortgage-free house worth €80,000. The house is in a nice neighborhood, and you believe that it should increase in value faster than inflation. Unfortunately, the house needs repairs. If rented today, the house would bring in €600 monthly, but if updates and repairs were made, the house would rent for €800 per month. To finance the repairs, you’ll need to take out a mortgage on the property. You would:

a. Sell the house

b. Rent the house as is

c. Remodel and update the house, and then rent it

In your opinion, is it more important to be protected from rising consumer prices (inflation) or to maintain the safety of your money from loss or theft?

a. Much more important to secure the safety of my money

b. Much more important to be protected from rising prices (inflation)

You’ve just taken a job at a small fast-growing company. After your first year, you are offered the following bonus choices. Which one would you choose?

a. A five-year employment contract

b. A €25,000 bonus

c. Stock in the company currently worth €25,000 with the hope of selling out later at a large profit

Some experts are predicting prices of assets such as gold, jewels, collectibles, and real estate (hard assets) to increase in value; bond prices may fall, however, experts tend to agree that government bonds are relatively safe. Most of your investment assets are now in high-interest government bonds. What would you do?

a. Hold the bonds

b. Sell the bonds, put half the proceeds into money market accounts, and the other half into hard assets

c. Sell the bonds and put the total proceeds into hard assets

d. Sell the bonds, put all the money into hard assets, and borrow additional money to buy more

Assume you are going to buy a home in the next few weeks. Your strategy would probably be:

a. To buy an affordable house where you can make monthly payments comfortably

b. To stretch a bit financially to buy the house you really want

c. To buy the most expensive house you can qualify for

d. To borrow money from friends and relatives so you can qualify for a bigger mortgage

Given the best and worst case returns of the four investment choices below, which would you prefer?

a. €200 gain best case; €0 gain/loss worst case

b. €800 gain best case; €200 loss worst case

c. €2,600 gain best case; €800 loss worst case

d. €4,800 gain best case; €2,400 loss worst case

Assume that you are applying for a mortgage. Interest rates have been coming down over the past few months. There’s the possibility that this trend will continue. But some economists are predicting rates to increase. You have the option of locking in your mortgage interest rate or letting it float. If you lock in, you will get the current rate, even if interest rates go up. If the rates go down, you’ll have to settle for the higher locked-in rate. You plan to live in the house for at least three years. What would you do?

a. Definitely lock in the interest rate

b. Probably lock in the interest rate

c. Probably let the interest rate float

d. Definitely let the interest rate float

In addition to whatever you own, you have been given €1,000. You are now asked to choose between:

a. A sure gain of €500

b. A 50% chance to gain €1,000 and a 50% chance to gain nothing

In addition to whatever you own, you have been given €2,000. You are now asked to choose between:

a. A sure loss of €500

b. A 50% chance to lose €1,000 and a 50% chance to lose nothing

Suppose a relative left you an inheritance of €100,000, stipulating in the will that you invest ALL the money in ONE of the following choices. Which one would you select?

a. A savings account or money market mutual fund

b. A mutual fund that owns stocks and bonds

c. A portfolio of 15 common stocks

d. Commodities like gold, silver, and oil

If you had to invest €20,000, which of the following investment choices would you find most appealing?

a. 60% in low-risk investments, 30% in medium-risk investments, 10% in high-risk investments

b. 30% in low-risk investments, 40% in medium-risk investments, 30% in high-risk investments

c. 10% in low-risk investments, 30% in medium-risk investments, 60% in high-risk investments

Your trusted friend and neighbor, an experienced geologist, is putting together a group of investors to fund an exploratory gold mining venture. The venture could pay back 50 to 100 times the investment if successful. If the mine is a bust, the entire investment is worthless. Your friend estimates the chance of success is only 20%. If you had the money, how much would you invest?

a. Nothing

b. One month’s salary

c. Three month’s salary

d. Six month's salary

**Supplementary Figure**


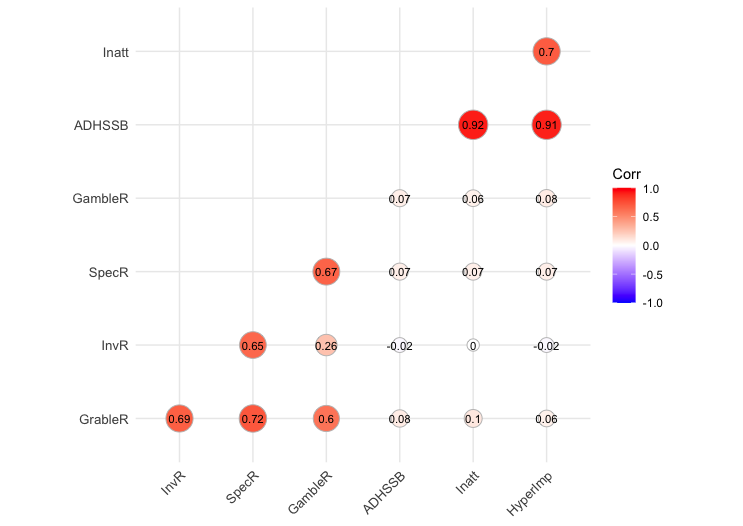


**Supplementary Figure 1:** Cell colors indicate correlation coefficients. Abbreviations: Corr = correlation coefficient; ADHSSB = total ADHD score; Inatt = inattention subscale; HyperImp = hyperactivity/impulsivity subscale; GrableR = total score of Grable Risk Tolerance Scale; SpecR = speculative risk; GambleR = gambling risk; InvR = investment risk.

**Supplementary Table**

|  | **FRT** | **ADHS-SB** | **Age** | **Education** | **Income** | **Assets** |
| --- | --- | --- | --- | --- | --- | --- |
| **FRT** | 1.0*** | 0.002 | -0.093** | -0.129*** | -0.072* | -0.007 |
| **ADHS-SB** | 0.002 | 1.0*** | -0.119*** | -0.103*** | -0.139*** | -0.226*** |
| **Age** | -0.093** | -0.119*** | 1.0*** | 0.261*** | 0.45*** | 0.538*** |
| **Education** | -0.129*** | -0.103*** | 0.261*** | 1.0*** | 0.513*** | 0.283*** |
| **Income** | -0.072* | -0.139*** | 0.45*** | 0.513*** | 1.0*** | 0.585*** |
| **Assets** | -0.007 | -0.226*** | 0.538*** | 0.283*** | 0.585*** | 1.0*** |

**Supplementary Table 1:** Spearman correlations between FRT, ADHD-SB, and demographics; **p* < .05, ***p < .01 , ***p < .001*; Education/Income/Assets: 1 = low, 5 = high.
